# Promoting *in vivo* remyelination with small molecules: a neuroreparative pharmacological treatment for Multiple Sclerosis

**DOI:** 10.1038/srep43545

**Published:** 2017-03-03

**Authors:** Eva María Medina-Rodríguez, Ana Bribián, Amanda Boyd, Valle Palomo, Jesús Pastor, Alfonso Lagares, Carmen Gil, Ana Martínez, Anna Williams, Fernando de Castro

**Affiliations:** 1 Grupo de Neurobiología del Desarrollo-GNDe, Hospital Nacional de Parapléjicos, Finca la Peraleda s/n, E- 45071, Toledo, Spain; 2 Instituto Cajal-CSIC, Avda. Dr. Arce 37, E-28002, Madrid, Spain; 3 MRC-Centre for Regenerative Medicine, University of Edinburgh, 5 Little France Drive, EH164UU, Edinburgh, UK; 4 Centro de Investigaciones Biológicas, CIB-CSIC, Calle Ramiro de Maeztu 9, E-28040, Madrid, Spain; 5 Servicio de Neurofisiología Clínica, Hospital La Princesa, Calle Diego de León 62, E-28006,Madrid, Spain; 6 Servicio de Neurocirugía, Hospital 12 de Octubre, Avda. de Córdoba s/n, E-28041,Madrid, Spain

## Abstract

Multiple Sclerosis (MS) is a neurodegenerative disease where immune-driven demyelination occurs with inefficient remyelination, but therapies are limited, especially those to enhance repair. Here, we show that the dual phosphodiesterase (PDE)7- glycogen synthase kinase (GSK)3 inhibitor, VP3.15, a heterocyclic small molecule with good pharmacokinetic properties and safety profile, improves *in vivo* remyelination in mouse and increases both adult mouse and adult human oligodendrocyte progenitor cell (OPC) differentiation, in addition to its immune regulatory action. The dual inhibition is synergistic, as increasing intracellular levels of cAMP by cyclic nucleotide PDE inhibition both suppresses the immune response and increases remyelination, and in addition, inhibition of GSK3 limits experimental autoimmune encephalomyelitis in mice. This combination of an advantageous effect on the immune response and an enhancement of repair, plus demonstration of its activity on adult human OPCs, leads us to propose dual PDE7-GSK3 inhibition, and specifically VP3.15, as a neuroprotective and neuroreparative disease-modifying treatment for MS.

In the Central Nervous System (CNS), demyelinating diseases, such as Multiple Sclerosis (MS), result in devastating long-term neurological damage. MS is a chronic autoimmune and neurodegenerative disease characterized by inflammation, oligodendrocyte loss, demyelination and axonal damage. Current MS licensed treatments are immunomodulatory, reducing the number of relapses, but with no effect on the accumulation of disability in progressive MS^1^. As progressive disability is thought to be secondary to irreversible neurodegeneration, neuroprotective therapies are being sought as a new group of MS therapies^2^. One of the most effective ways of enhancing neuroprotection is to improve remyelination, a spontaneous process by which demyelinated axons undergo ensheathment with new myelin sheaths, and this restores metabolic support and fast conduction of nerve impulses. Spontaneous remyelination is mediated by SVZ stem cells and endogenous oligodendrocyte progenitor cells (OPCs) present throughout the adult CNS that differentiate into mature myelinating oligodendrocytes^3^,^4^,^5^,^6^,^7^,^8^,^9^,^10^,^11^. Following demyelinating damage, adult SVZ stem cells can mobilize and participate in remyelination as shown in several animal models of demyelination^12^,^13^,^14^,^15^,^16^,^17^. In addition, in mammals like rodents and human, the presence of adult endogenous OPCs, which represent approximately 8–9% of the total population of the white matter of an adult brain and 2–3% of the gray matter^18^,^19^,^20^,^21^,^22^, replace oligodendrocytes in physiological myelin turnover^4^,^23^,^24^, and react in response to a variety of pathologies^25^,^26^,^27^,^28^. However, remyelination mediated by these adult endogenous OPCs is inefficient and ultimately incomplete in MS patients, at least in part due to failure of adequate OPC differentiation into myelinating oligodendrocytes. This has focused our efforts on discovering and developing factors/drugs that enhance OPC maturation and subsequent remyelination for translation into therapies.

In this context, we have recently shown the anti-inflammatory and neuroprotective effects of the cAMP-specific phosphodiesterase 7 (PDE7) inhibitors in animal models of spinal cord injury, stroke, Parkinson´s and Alzheimer´s diseases, and MS^29^,^30^,^31^,^32^,^33^,^34^,^35^,^36^,^37^,^38^. Although their effect on remyelination remains unknown, previous data from our group have shown that PDE7 inhibitors favour the differentiation and survival of mouse cortical OPCs and the differentiation of adult human OPCs *in vitro*^39^. PDE7 inhibition leads to an enhancement of the intracellular levels of cAMP without intolerable gastrointestinal side effects^40^, making them potential and attractive therapeutic agents. Furthermore, glycogen synthase kinase 3 (GSK3) inhibition reduces the number of Th17 and Th1 cells with consequent alleviation of experimental autoimmune encephalomyelitis (EAE)^41^,^42^.

In the present work, we show that allosteric modulation of both PDE7 and GSK-3, using 5-imino-1,2,4-thiadiazoles: (i) reduces symptoms in the EAE animal model of demyelination (as previously reported^36^) and, (ii) enhances remyelination in two other demyelinating mouse models where demyelination occurs with minimal adaptive immune system contribution (using lysophosphatidylcholine-LPC- or cuprizone). Our results lead us to propose these dual PDE7-GSK3 inhibitors, especially VP3.15 with its good oral bioavailability and CNS penetration, as potential combined anti-inflammatory and pro-remyelinating therapies for MS.

## Results

### Dual PDE7-GSK3 inhibition by VP3.15 enhances murine and adult human OPC differentiation without affecting their survival or proliferation

**Figure 1:**
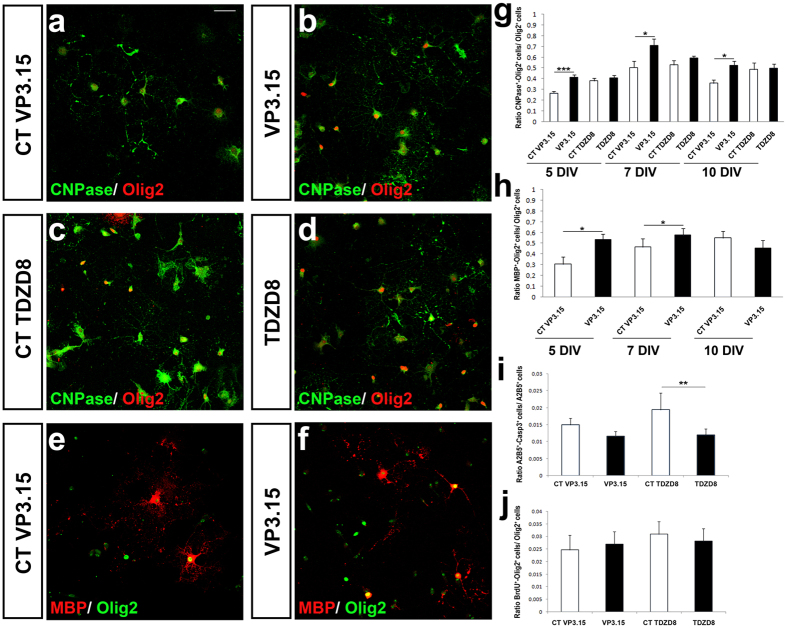
VP3.15 favors differentiation of murine OPCs without affecting their proliferation. (**a**–**d**) Immunofluorescence images showing the expression of Olig2 and CNPase by differentiated OPCs from P0 mice after 5 DIV cultured in the presence of VP3.15 (**a**,**b**) or TDZD8 **(c,d)**. (**g**) Quantification of CNPase^+^-Olig2^+^ cells with respect to the total number of Olig2^+^ cells at 5, 7 and 10 DIV. The presence of VP3.15 increases the number of oligodendrocytes in comparison with control conditions. TDZD8 did not induce changes in OPC differentiation. **(e**,**f)** Immunofluorescence images showing the expression of Olig2 and MBP by differentiated OPCs from P0 mice after 5DIV, cultured in the presence of VP3.15. **(h)** Quantification of MBP^+^-Olig2^+^ cells with respect to the total number of Olig2^+^ cells at 5, 7 and 10 DIV in the presence of VP3.15. **(i)** Determination of apoptotic oligodendroglia (Casp3^+^-A2B5^+^ cells). OPC survival was enhanced in the presence of TDZD8 only (1 μM). **(j)** Quantification of BrdU incorporation by double immunocytochemistry on OPCs from P0 mice. The presence of VP3.15 or TDZD8 did not modify the number of BrdU^+^-Olig2^+^ cells compared with their respective controls. Scale bar represents 25 μm for (**a–f** ). Values are given as mean ± SEM and the results of Student’s *t*-test are represented as *p < 0.05, **p < 0.01, and ***p < 0.001.

Previously, we have shown that PDE7 is expressed in OPCs and that PDE7 inhibitors (including VP1.15) enhance OPC differentiation and survival *in vitro*^39^. However, the dual PDE7-GSK3 inhibitor VP3.15 was designed and synthesized as a drug-like molecule with a better safety profile, and improved pharmacodynamic and pharmacokinetic properties^36^,^43^ (Tables 1 and 2). It shows oral bioavailability and brain penetration in mice after oral and i.p. administration (Table 2). We determined whether the effects seen on OPCs could be reproduced with this new inhibitor, VP3.15. Addition of VP3.15 to the culture medium of OPCs isolated from P0 mice led to a significant increase in the number of mature oligodendrocytes (identified as CNPase^+^-Olig2^+^ and MBP^+^-Olig2^+^ double positive cells) in comparison to control cultures after 5, 7 and 10 days *in vitro* (DIV; Fig. 1a,b,e–h). However, VP3.15 had no additional effect on morphology of mature oligodendrocytes as the number of processes and subprocesses was not different to control (number of primary cytoplasmic processes: 4.9 ± 0.35 in the control group vs. 4.7 ± 0.31 in cells treated with VP3.15; Student´s *t*-test: p = 0.64; number of cytoplasmic subprocesses 8.588 ± 0.875 in the control group vs. 8.111 ± 0.970 cells treated with VP3.15, Student’s *t*-test: p = 0.62).

VP3.15 is a dual inhibitor of PDE7 and GSK3^33^,^36^,^43^ and it is already known that GSK3 inhibition improves oligodendrocyte survival, differentiation and myelination^44^. To distinguish if any observed effects were specific to either PDE7 or GSK3 inhibition, we used a GSK3-specific inhibitor (TDZD8), which have previously shown does not inhibit PDE7^33^. In the presence of TDZD8, no effect on OPC differentiation was shown (Fig. 1c,d,g) clarifying that GSK3 inhibition itself did not influence OPC differentiation, at least in the current experimental conditions. However, TDZD8 increased OPC survival (Fig. 1i). There were no changes in OPC proliferation in the presence of either VP3.15 or TDZD8 (Fig. 1j).

**Figure 2:**
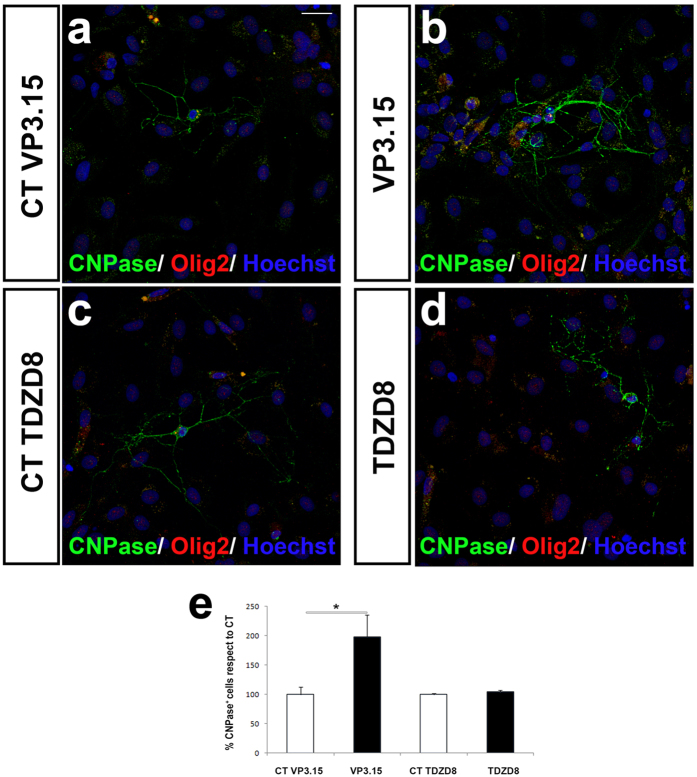
VP3.15 favors differentiation of adult human OPCs. **(a–d)** Immunofluorescence images for CNPase and Olig2 labelling onadult OPCs from human cerebral cortex, after 5 DIV in the presence of VP3.15 **(a,b)** or TDZD8 (1 μM) **(c,d)**. (**e**) The quantification of CNPase^+^ cells in human cultures showed that in the presence of the VP3.15 OPC differentiation was higher than in control conditions, while with TDZD8 there was no effect. Scale bar represents 25 μm for (**a–d**). Values are given as mean ± SEM and the results of Student’s *t*-test are represented as *p < 0.05, **p < 0.01, and ***p < 0.001.

Adult human OPCs isolated from non-tumoral neurosurgery samples (see Methods) also showed increased differentiation in the presence of VP3.15. As in the case of murine OPCs, the number of CNPase^+^-Olig2^+^ cells significantly increased after 5 DIV with the PDE7 inhibitor compared to control values (Fig. 2a,b,e). As in murine cultures VP3.15 had no additional effect on morphology of mature oligodendrocytes as the number of processes and subprocesses was not different to control (number of primary cytoplasmic processes: 2.9 ± 0.21 in the control group vs. 2.75 ± 0.20 in cells treated with VP3.15; Student´s *t*-test: p = 0.72; number of cytoplasmic subprocesses 4.3 ± 0.2 in the control group vs. 4.8 ± 0.43 cells treated with VP3.15, Student´s *t*-test: p = 0.83). Again, the GSK3 inhibitor TDZD8 did not show any effect on differentiation of adult human OPC cultures (Fig. 2c–e).

### Dual PDE7-GSK3 inhibition enhances remyelination in cerebellar slice cultures

**Figure 3:**
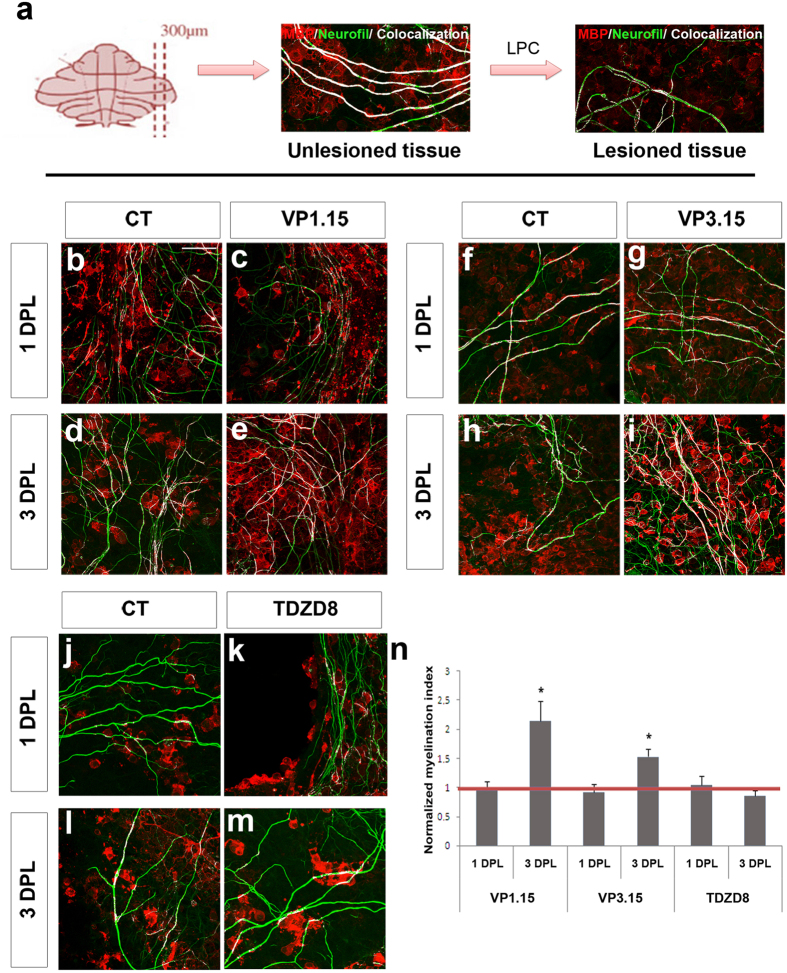
PDE7-GSK3 inhibition favors remyelination after demyelination by LPC in cerebellar slices. (**a**) Immunofluorescence images showing cerebellar slices with or without treatment with LPC (0.5 mg/ml). Untreated tissue shows most axons (green) wrapped by oligodendrocytes (red). After induction of demyelination with LPC, most oligodendrocytes were lost. (**b**–**m**) Images show tissue after 1 and 3 days post-lesion (DPL) where oligodendrocytes (red) and axons (green) can be observed and the overlapping areas (white) show myelinated fibres after treatment with 5 μM of VP1.15 **(b**–**e)**, VP3.15 **(f**–**i)** or TDZD8 **(j–m**). **(n)** Plot showing the normalized myelination index which represents the co-localization area with respect to the total area occupied by fibers and normalized with respect to the control values to which a value of 1 was assigned (red line). The treatment with inhibitors for 24 h (1DPL) did not induce an observable effect on demyelinated slices, showing that the baseline between conditions is similar, but when the treatment was extended for 48 more hours (3DPL) a positive effect on remyelination was observed withPDE7-GSK3 inhibition with both VP1.15 and VP3.15. TDZD8 showed no effect. Scale bar represents 25 μm for (**a–d**). Values are given as mean ± SEM and the results of Student’s t test are represented as *p < 0.05, **p < 0.01, and ***p < 0.001.

We then tested the effects of dual PDE7-GSK3 inhibition on *ex vivo* cultures from cerebellar slices demyelinated with LPC (lysophosphatidylcholine; see Methods). One day after the LPC lesion (1DPL), the axons had lost almost all myelin sheaths (labeled with an antibody against myelin basic protein-MBP) compared to non-damaged tissue (Fig. 3a). In these remyelination assays, we used the two related dual inhibitors of PDE7 and GSK3 enzymes, VP1.15 (as used previously in monocultures) and VP3.15 (as our new inhibitor), with TDZD8 as a control for testing GSK3 inhibition alone (see above). As early as 3 days of treatment after demyelination, remyelination was increased under treatment with either dual inhibitor (VP1.15, Fig. 3d,e,n; VP3.15, Fig. 3h,i,n), but not with the GSK3 inhibitor (TDZD8, Fig. 3l–n). No differences in remyelination were observed at baseline, one day after treatment (Fig. 3b,c,f,g,j,k,n).

### Dual PDE7-GSK3 inhibition improves *in vivo* remyelination

**Figure 4:**
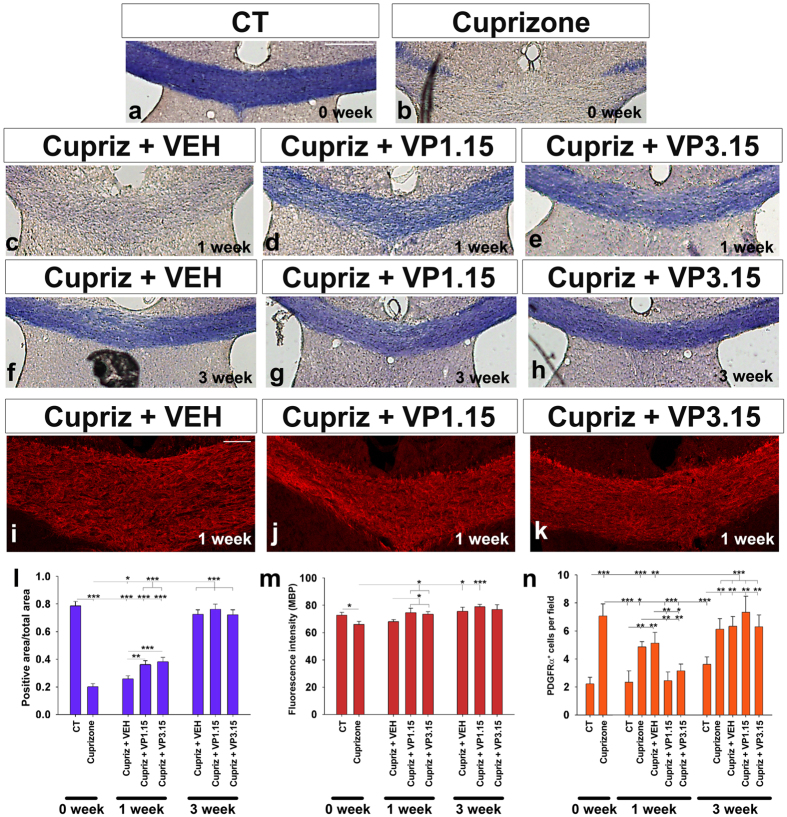
PDE7-GSK3 inhibition favors remyelination. **(a–h)** Eriochrome cyanine stained images of the corpus callosum ofadult mice. Images correspond to **(a)** control mice, **(b)** mice whose corpus callosum was demyelinated by cuprizone, **(c–h)** mice treated with different doses of PDE7-GSK3 inhibitors or vehicle after injury at 1 or 3 weeks. **(l)** The histogram shows the quantification of the eriochrome cyanine positive area respect to the total measured area. After cuprizone withdrawal (0 week), the midline of the corpus callosum was almost completely demyelinated **(a,b,l)**. 1 week after cuprizone withdrawal, mice treated with the vehicle did not show remyelination **(c,l)** while mice treated with VP1.15 or VP3.15 showed almost complete remyelination **(c,d,l)**. 3 weeks after cuprizone withdrawal, the vehicle-injected group and thePDE7-GSK3 inhibitors treated showed similar amounts of myelin **(f,g,h,l)**. **(i–k)** Images show immunofluorescence for MBP in1 week after cuprizone treatment withdrawal group. **(m)** The histogram shows MBP fluorescence intensity after cuprizone withdrawal and 1 and 3 weeks of PDE7-GSK3 inhibitor subsequent treatment. MBP staining decreased after cuprizone feeding, but recovered after PDE7-GSK3 inhibitor treatment. 3 weeks after no differences were found between groups. **(n)** Histogram shows the number of OPCs per field after 1 and 3 weeks inhibitors treatment. Scale bar represents 250 μm for (**a–h**), and 100 μm for (**i–k**). Values are given as mean ± SEM and the results of Student’s *t-*test are represented as *p < 0.05, **p < 0.01, and ***p < 0.001.

The cuprizone model of demyelination is a consistent and anatomically reproducible method which allows the study of remyelination in response to drugs after cuprizone withdrawal^45^,^46^. After 5 weeks of cuprizone diet, eriochrome-cyanine staining showed marked corpus callosal demyelination in comparison with control mice (Fig. 4a,b,l), in agreement with previous reports^47^,^48^. Mice were treated with cuprizone diet for 5 weeks, then the diet was withdrawn and treatment with a dual inhibitor started. One week after cuprizone withdrawal, the experimental groups treated with VP1.15 and VP3.15 (Fig. 4d,e,l) showed a significantly higher myelin staining (with eriochrome cyanine) than their corresponding control (vehicle-injected) group (Fig. 4c,l), reflecting the improvement of remyelination by dual PDE7-GSK3 inhibition *in vivo*. After treatment with our dual inhibitors for two more weeks (analysed 3 weeks after cuprizone withdrawal; see Methods), the amount of myelin in the corpus callosum was equivalent between treatment and vehicle-injected mice. These data reflect the almost very efficient complete spontaneous remyelination at this time point in this model at this age (8 week mice) (Fig. 4f–h,l). Myelin quantification by MBP immunodetection confirmed these results, with similar effects in both VP1.15 and VP3.15 treated groups (Fig. 4i–k,m). In addition, there were more oligodendrocytes in the lesioned area in the presence of either inhibitor compared to control, as seen by immunostaining using anti-CC1 antibodies (Supplementary Fig. 1a–d).

A higher number of OPCs (identified as PDGFRα^+^ cells) was found in the studied area after cuprizone compared to control animals (Fig. 4n). This number decreased one week after cuprizone withdrawal. Of note, mice treated with VP1.15 or VP3.15 showed a significantly lower number of OPCs one week after cuprizone withdrawal compared with the vehicle-injected mice (Fig. 4n). This decrease in OPC number overlaps with the increase in remyelination suggesting that OPCs recruited towards demyelinated lesions are already differentiating into mature oligodendrocytes to remyelinate the lesion. After 3 weeks of cuprizone withdrawal, myelin staining with both MBP and eriochrome cyainine, was similar in all groups (Fig. 4f–h,l,m). However, at 3 weeks, the number of OPCs in all of the groups fed cuprizone remained higher than controls without cuprizone, suggesting an increased response of OPCs to injury to levels beyond that required for complete remyelination (Fig. 4n).

In addition, immediately after cuprizone withdrawal (0 week), we observed a large increase in the number of activated microglia (Supplementary Fig. 2a,b,h), which declined in the first week after cuprizone withdrawal (Supplementary Fig. 2c,d,h). When treated with the heterocyclic compounds or the vehicle, firstly a reduced activation was observed in response to the vehicle injections alone (see Methods; Supplementary Fig. 2d,e,h). Neither VP1.15 nor VP3.15 induced changes in microglial activation with the experimental conditions used compared with the vehicle-injected group (Supplementary Fig. 2e–h). An increased level of astrocyte immunoreactivity was also observed in all the cuprizone-fed mice with respect to the non-cuprizone treated mice, and this astroglial reaction remained similar in the presence of the vehicle or the dual PDE7-GSK3 inhibitors (Supplementary Fig. 2i). All these results indicate a specific effect of dual PDE7-GSK3 inhibitors on OPCs.

**Figure 5:**
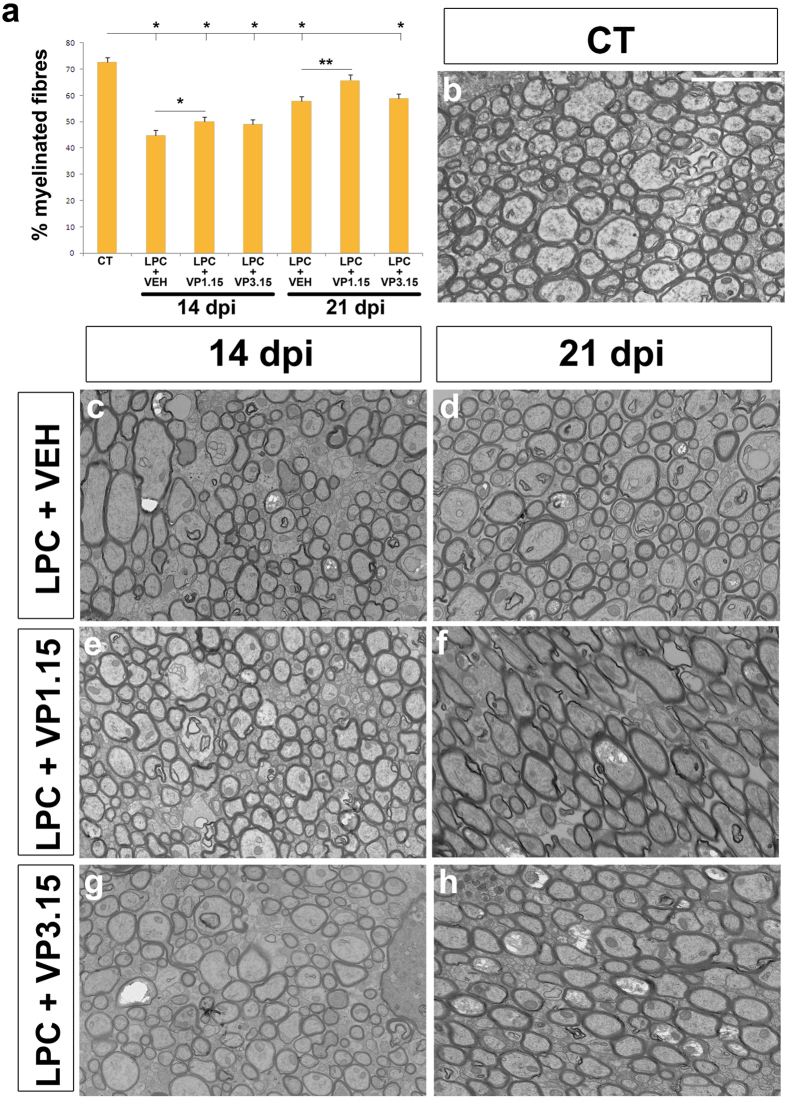
PDE7-GSK3 inhibition increased the percentage of myelinated fibres in the repair phase after LPC demyelination. **(a)** Histogram shows the percentage of myelinated axons in the corpus callosum in control and LPC lesioned mice, sacrificed 14 or 21 days post-injection (dpi). Mice were treated with either vehicle (LPC+VEH), or PDE7-GSK3 inhibitors VP1.15 (LPC+VP1.15) or VP3.15 (LPC+VP3.15) from day 9 post lesion. In the lesion site, the percentage of myelinated axons was increased in mice treated with VP1.15. Those that received three injections of this inhibitor from day 9 post lesion, analyzed at 21 dpi had a similar percentage compared to that observed in control unlesioned mice. **(b–h)** Electron microscopy images showing the ultrastructure of the corpus callosum of adult control uninjected mice **(b)** and the experimental groups: **(c,d)** LPC and vehicle **(e,f)**, LPC and VP1.15 **(g,h)** LPC and VP3.15. Mice perfused at 14 dpi were treated with two injections **(c,e,g)** while mice perfused 21 dpi received one more **(d,f,h)**. Statistical analysis was with One-way ANOVA and Tukey’s post hoc test (*p < 0.05) or Student’s *t*-test (or Mann–Whitney rank-sum test). Values are given as mean ± SEM and the results of Student’s *t*-test are represented as *p < 0.05, **p < 0.01, and ***p < 0.001. Scale bar represents 5 μm for (**a**–**g**).

**Figure 6:**
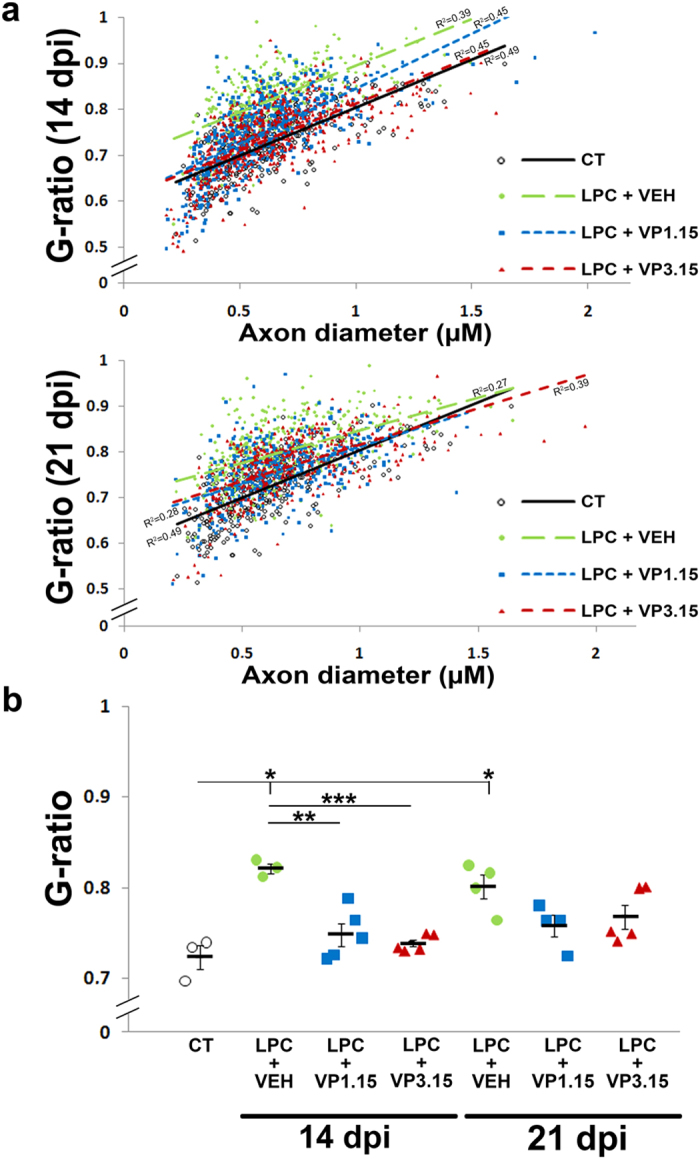
PDE7-GSK3 inhibition increased myelin thickness in the repair phase in the LPC demyelination model. **(a)** Dot plot of the G-ratio with respect to the axon diameter of each measured fibre. The lines of best fit show that the G-ratio was lower in mice treated with PDE7-GSK3 inhibitors (LPC+VP1.15 or LPC+VP3.15) compared to non-treated (LPC+VEH), i.e. they have thicker myelin sheaths, and these values are close to that of control unlesioned mice. **(b)** The plot represents the averages of the G-ratios for each mouse and the average for each group showing an increase of myelin thickness (decrease of G-ratio) in mice treated with PDE7-GSK3 inhibitors compared to non-treated groups (LPC+VEH). The myelin thickness in treated mice approached but never reached the values of G-ratio of control mice (CT) thus showing no hypermyelination. The comparison of each group with control was made using One-way ANOVA and Tukey’s post hoc test (*p < 0.05) and treated groups were compared with groups injected with the vehicle using a Student’s t test (or Mann–Whitney rank-sum test). Values are given as mean ± SEM and the results of Student’s *t*-test are represented as *p < 0.05, **p < 0.01, and ***p < 0.001.

Finally, we used injection of LPC to induce demyelination *in vivo*, which allowed us to study the direct effect of drugs on spontaneous remyelination in focal lesions with a minimal participation of the adaptive immune response. To measure remyelination, we examined the percentage of axons in the lesion that were myelinated and the thickness of the myelin sheath by electron microscopy, as remyelinated fibers typically have thinner myelin sheaths^49^,^50^. As the mode of action of these drugs is enhanced differentiation of OPCs, we treated mice 9 days after demyelination, when OPCs have been recruited to the demyelinated lesion, before normal differentiation occurs. With this treatment paradigm, we saw an increase of the percentage of myelinated callosal fibers in the mice treated with VP1.15 (LPC+VP1.15), at 14 or 21 dpi when compared to mice treated with the vehicle (LPC+VEH; Fig. 5a,c–f). However, treatment with VP3.15 (LPC+VP3.15) did not show any effect (Fig. 5a,g,h). In the case of VP1.15, mice perfused at 21 dpi had a similar percentage of myelinated axons to that observed in normal control (uninjected) mice (CT; Fig. 5b), which suggests an almost complete remyelination at this time-point (Fig. 5a,b,f). In addition, both treatments (LPC+VP1.15 and LPC+VP3.15) increased the thickness of myelin sheaths compared to those in untreated demyelinated control mice (Fig. 6a,b). In fact, myelin thickness of treated mice was close to that observed in control mice (CT), but with no sign of hypermyelination (Fig. 6b).

## Discussion

Here, we show that dual PDE7-GSK3 inhibition aids CNS remyelination in a variety of *ex vivo* and *in vivo* demyelination models, by enhancing differentiation of OPCs. We found the compound VP3.15 to be effective at enhancing speed of remyelination in the cuprizone model but it showed no significant effect in the focal LPC-induced demyelination model. This may reflect differences in the toxins used to cause demyelination, as cuprizone is known to cause global demyelination with axonal injury, compared to a focal injury with little axonal damage using LPC. Therefore the most likely explanation is that as both of these models remyelinate very effectively, with a more extensive injury, both in severity and extent, accelerated remyelination may be easier to identify. This could be tested in old mice, that remyelinate slower, seeking enhancers of remyelination in future studies.

In a prior work, we have shown the beneficial effect of inhibition of these pathways in T-cell driven models of demyelination^36^, suggesting that these small molecules may be useful to aid both the inflammatory T-cell driven relapsing-remitting (RR) phase of MS and the neurodegenerative progressive phase of the disease. If this holds true in human clinical trials, then this presents a novel beneficial therapy for MS. Disappointingly, drugs useful in RRMS to date have failed to have a positive effect in progressive MS^51^,^52^,^53^,^54^, leading to the view that we will need a combinatorial approach with separate classes of drugs for the RR phase and the progressive phases of MS. Dual PDE7-GSK3 inhibition perhaps provides this combinatorial approach in one drug. Recovery in the neurological score in EAE after treatment with the dual inhibitor VP3.15 shows similar effectiveness to the now licensed MS immunomodulatory drug fingolimod, both when administered in a preventive or treatment regime^55^. Dual PDE7-GSK3 inhibition enhances remyelination (and thus neuroprotection) by enhancing OPC differentiation. However, inhibition of GSK3 alone, with TDZD8, shows no effects on OPC proliferation or differentiation, in spite of improved OPC survival. Previous results exclusively targeting inhibition of PDE7 with the quinazoline TC3.6, enhances OPC differentiation less efficiently than the dual inhibitor VP1.15^39^. The mechanism of action of dual PDE7-GSK3 inhibition on remyelination is likely specific to OPCs, as treatment did not modify microglial or astrocyte number, at least in the cuprizone *in viv*o model. The increase of remyelination in both slice models and *in viv*o was fast in rodents: an effect was seen in merely 3 days in slices and 7 days *in vivo*. Logic (though no experimental evidence) dictates that faster remyelination in humans (as well as more efficient extent of remyelination) will be beneficial, reducing axon vulnerability. Combination of these drugs with others promoting the effective recruitment of OPCs towards demyelinating lesions may provide an even greater advantage^56^.

As potential MS therapies, these dual PDE7-GSK3 inhibitors are orally bioavailable and readily penetrate the blood-brain-barrier, one of the major concerns regarding the potential pro-remyelination therapy anti-LINGO antibody BIIB033^2^,^57^. The pharmacological properties of VP3.15 are particularly encouraging for further development as a drug, though both VP1.15 and VP3.15 will be tested in regulatory toxicology studies, including chemical, pharmaceutical technology and toxicological ICH developments, with the aim that the drug with the better therapeutic window will be taken to clinical trial. Also, unlike antibody treatments, these are not expected to provoke immune reactions^58^. Some cAMP specific PDE inhibitors have already been approved for use in other diseases – e.g. apremilast. The PDE7-GSK3 inhibitors used here (VP3.15 and VP1.15) have previously also shown an immunomodulatory effect in spinal cord injury models (as well as EAE)^34^,^36^, without emetic side effects^36^,^40^.

To date, several other pathways and drug targets have been shown to improve remyelination based on evidence from a variety of animal models (reviewed in refs 59, 60, 61). Some have even been tested on human OPCs derived from ES cells^62^,^63^,^64^ but few of them have been tested on adult human OPCs, which may well behave differently, as spontaneous remyelination decreases with age^65^. There are just three published studies demonstrating the myelination capability of adult human OPCs transplanted into *ex vivo* and *in vivo* models^66^,^67^,^68^. The pro-myelinating drugs now in trial (anti-LINGO antibodies BIIB033, rHIgM22, clemastine, quetiapine, GSK239512, domperidone, VX15, olexisome and GNbAC1^69^,^70^,^71^,^72^,^73^,^74^,^75^; for a recent review on the subject, see ref. 61) have not been studied for their effects in human adult OPCs, representing a translational risk due to putative differences in the biology and physiology of this cell type. Whether this risk is justified will be revealed with the future results of the on-going studies.

Therefore, the combination of GSK3 and PDE7 inhibition may synergistically activate beneficial anti-inflammatory and pro-remyelination pathways with potential high therapeutic value. Only clinical trials will confirm the real value of these small molecules as efficient disease-modifying and neuroprotective drugs for MS patients, but their pharmacological qualities in rodents and effects on adult human OPCs make them promising.

## Methods

### Animals

P0 and P7 CD1 mice were obtained from Charles River Laboratories (Wilmington, MA, USA) and maintained in the animal facilities at the Hospital Nacional de Parapléjicos (Toledo, Spain).

8 weeks old C57/BL6 male mice for demyelinating models were obtained from Charles River Laboratories (Wilmington, MA, USA) and maintained in the animal unit of MRC Centre for Regenerative Medicine, (University of Edinburgh, Edinburgh, UK) or in the animal facilities at the Hospital Nacional de Parapléjicos (Toledo, Spain).

All animal experiments were carried out in accordance with Spanish (RD223/88) and European (2010/63/EU) regulations, and they were approved by the Animal Review Board at the Hospital Nacional de Parapléjicos (agreement number: SAPA001) or in accordance with the University of Edinburgh regulations under Home Office rules, with local ethics committee consent.

### Human samples

Human brain samples were obtained from adult patients of temporal lobe epilepsy resistant to pharmacological treatment or traumatic brain injury; they underwent surgical treatment by the Neurosurgery Service at the Hospital Universitario de La Princesa (Madrid, Spain) and Hospital 12 de Octubre (Madrid, Spain). All tissue samples were obtained under protocols approved by the Research Ethics Committee of Toledo (Spain) review boards. All were conducted in accordance with the Helsinki Declaration and were approved by the Research Ethics Committee of Toledo (Spain). All patients provided informed consent prepared under the guidelines of the Research Ethics Committee at their respective hospital in Madrid (Spain).

### PDE7-GSK3 inhibitors

VP1.15, VP3.15 and TDZD8 (Table 1) were synthesized at Dr. Ana Martinez´s Lab (Centro de Investigaciones Biológicas-CIB-CSIC) following published procedures^43^,^76^.

### Cell culture

We isolated OPCs, as previously described^39^,^77^. Briefly, a stock papain solution was prepared containing (0.9 g/ml; Worthington Biochemical), L-cysteine (0.2 mg/ml; Sigma), and EDTA (0.2 mg/ml; Sigma) in Hank’s Balanced Salt Solution (HBSS; Invitrogen) without Ca^2+^ and Mg^2^. Then, the cerebral cortices of P0 CD1 mice were dissected out and placed for 5 minutes at 37 °C in a medium containing the stock papain solution in HBSS with Ca^2+^ and Mg^2+^ (1:10; Invitrogen) in order to easily remove the meninges. Once meninges and choroid plexus were removed, the tissue was enzymatically dissociated for 5 minutes at 37 °C again by using the papain solution (1:10 in HBSS without Ca^2+^ and Mg^2+^) and the digested tissue was then filtered through a 100 μm nylon mesh strainer (BD Biosciences) and seeded on poly-L-ornithine-coated (Sigma) 75 cm^2^ flasks in DMEM medium (Invitrogen) containing 10% foetal bovine serum (FBS; BioWhittaker) and 100 U/mL penicillin, 0.1 mg/mL streptomycin and 0.25 μg/ml Amphotericin B antibiotic antimycotic solution (Sigma). Cultures were maintained at 37 °C and 5% CO_2_, and the medium was changed every 3 days. When the cultures reached confluence, they were shaken overnight at 250 rpm and at 37 °C in order to detach the OPCs located on the top of the confluent astrocyte monolayer and the medium was then filtered through a 40μm nylon mesh strainer (BDBiosciences) and centrifuged at 900 rpm. Cells were seeded twice (45 minutes each) in bacterial grade Petri dishes (Sterilin) to remove microglial cells. The resulting enriched oligodendrocyte progenitor cell suspension was counted and seeded.

For cells obtained from human biopsies, the same protocol was used except that papain solution was used 1:2.5 in HBSS for 10 minutes for meningeal disintegration and 1:10 in HBSS for 15 minutes for the tissue digestion, 25 cm^2^ flasks were used, the medium was supplemented with 10 ng/ml of human PDGF-AA (Millipore) for 2 weeks and then incubated for 2 more weeks without growth factors before shaking and the shaking was carried out at 230 rpm instead of 250 rpm.

### Survival assay

Purified OPCs were placed on coverslips coated with poly-L-lysine (Sigma) and laminin (Engelbreth-Holm-Swarm murine sarcoma; Sigma; 2 × 10^4^ cells/well) and they were cultured with dual PDE7-GSK3 or GSK3 inhibitors (VP3.15 or TDZD8; 1 μM) in a previously described serum-free differentiation medium^78^, consisting of BME:F12 (1:1; Invitrogen) supplemented with 100 μg/ml transferring (Sigma), 20 μg/ml putrescine (Sigma), 12.8 ng/ml progesterone (Sigma), 10.4 ng/ml sodium selenite (Sigma), 25 μg/ml insulin (Sigma), 0.8 μg/ml thyroxine (Sigma), 0.6% glucose (Normapur), 6.6 mM glutamine (Invitrogen) and 100 U/mL penicillin, 0.1 mg/ml streptomycin and 0.25 μg/ml Amphotericin B antibiotic anti-mycotic solution (Sigma). After 2 DIV, they were fixed with 4% paraformaldehyde (PFA) and subjected to immunocytochemistry for active caspase 3 (Casp3; 1:200; Abcam; Cat#ab13847) and the OPC marker A2B5 (1:10; Hybridoma Bank). 10–20 microphotographs from each coverslip were taken randomly with an In Cell Analyzer 1000 (GE-HealthCare) and the number of Casp3^+^-A2B5^+^ double positive cells was counted using the software In Cell Analyzer 1000 Workstation (GE-HealthCare). Data were expressed as a mean ratio of double positive cells for Casp3 and A2B5 with respect to the total number of A2B5^+^ cells ± SEM for each condition in at least three independent experiments.

### Proliferation assay

OPCs were placed on coverslips coated with poly-L-lysine (Sigma) and laminin (Sigma; 2 × 10^4^ cells/well) and they were incubated with the inhibitors (VP3.15 or TDZD8; 1 μM) in the serum free medium described above. After 42 h in culture, BrdU (50 μM; Sigma) was added for 6 h and after a total of 72 h in culture, the cells were fixed and BrdU incorporation was detected by immunocytochemistry (1:20; G3G4, HybridomaBank) combined with the detection of the oligodendroglial marker Olig2 (1:250; Millipore; Cat#AB9610). After immunostaining, coverslips were examined with an In Cell Analyzer1000 (GE-HealthCare) and 10–20 microphotographs from each one were taken randomly. The number of BrdU^+^-Olig2^+^ double positive cells was counted using the software In Cell Analyzer 1000 Workstation (GE-HealthCare) and the data were expressed as a mean ratio of BrdU^+^-Olig2^+^ double positive cells with respect to the total number of Olig2^+^ cells ± SEM, in at least three independent experiments.

### Differentiation assay

Purified OPCs were placed on coverslips coated with poly-L-lysine and laminin in 24-well tissue culture dishes at a density of 2 × 10^4^ cells/well. To promote differentiation, the cells were maintained in the differentiation medium (described above) and PDE7-GSK3 or GSK3 inhibitors (VP3.15 or TDZD8; 1 μM) were added to the culture medium. Different controls were used for the dual or single inhibitors due to the active substance being dissolved in differing amounts of DMSO. After 5, 7 and 10 DIV the cells were fixed with 4% PFA for further immunocytochemical analysis. Anti-CNPase antibodies (1:200;Covance; Cat#SMI-91R), anti-MBP antibodies (1:200; Serotec; Cat#MCA4095) for detecting oligodendrocytes and anti-Olig2 antibodies (1:250; Millipore; Cat#AB9610) as a marker for all oligodendroglial stages were used. Ten random fluorescence digital images were taken in each coverslip with a DFC480 FX digital camera (Leica) coupled to a Leica DM5000 B microscope for quantification. Data were expressed as a mean ratio of differentiated oligodendrocytes (CNPase^+^-Olig2^+^ double positive cells) with respect to total Olig2^+^ cells ± SEM or percentage of CNPase^+^ cells respect to control ± SEM, in at least three independent experiments, except with human samples (two independent experiments).

### Slice culture and quantification of *ex vivo* remyelination

The protocol used was modified from previously described 79. CD1 P7 mice were decapitated, and their cerebella were dissected into ice-cold HBSS (Invitrogen). 300 μm sagittal slices of cerebellum were cut using a McIllwain tissue chopper, placed on Millicell-CM organotypic culture inserts (3 slices per insert; Millipore) in a medium containing HBSS:BME: horse serum (1:2:1, Invitrogen), 28 mM D (+) - glucose (Normapur), 0.25 μM L-Glutamine (Invitrogen) and 100 U/mL penicillin, 0.1 mg/mL streptomycin and 0.25 μg/ml Amphotericin B antibiotic anti-mycotic solution (Sigma) and maintained at 5% CO_2_ and 37 °C. Medium was changed every two days, and after 7 days in culture, demyelination was induced by addition of 0.5 mg/ml LPC (Sigma) to the medium for 15 h. After that, slices were treated with fresh medium containing PDE7-GSK3 or GSK3 inhibitors (VP1.15, VP3.15 or TDZD8; 5 μM). Cultures were maintained for 1 or 3 days post lesion (DPL), and then fixed for 40 minutes and immunostained with anti-MBP antibodies (myelin basic protein –as a marker of myelin; 1:200; Serotec; Cat#MCA4095) and anti-neurofilament antibody (as a marker of axons; 1:200; Abcam; Cat#ab8135). Fluorescence images were taken on a confocal SP5 resonant scanner (Leica Microsystems) at 1μm intervals in white matter areas at x40 magnification. The photographs were merged and co-localisation of both markers was quantified with Image J software using the colocalization highlighter plugin. This measurement was related to the area occupied by axons (neurofilament staining) and normalized with control values showing the data as the mean normalized myelination index ± SEM. Quantification was made in at least three independent experiments.

### *In vivo* remyelination study in cuprizone demyelination model

The protocol used was modified from previously described^80^. Demyelination was induced by feeding 8 weeks old male C57/BL6 mice *ad libitum* with 0.2% cuprizone (bis-cyclo-hexanone-oxaldihydrazone; Sigma) mixed with milled chow (Altromin) for 5 weeks. A control group was maintained in the same conditions but fed with the chow without cuprizone. After cuprizone withdrawal, mice were intraperitoneally injected on 3 alternate days with PDE7-GSK3 inhibitors (VP1.15 or VP3.15; 10 mg/kg of animal) using a solution of the compound (100 mg/mL in DMSO) diluted 1:50 in a solution of 5% Tocrisolve (Tocris, UK) in distilledwater^36^ or with the vehicle. 1 week after the cuprizone withdrawal, mice were transcardially perfused with 4% PFA in phosphate buffer. Another group was maintained for 3 weeks after cuprizone withdrawal and injected with 3 doses per week on alternate days of the PDE7-GSK3 inhibitors (10 mg/kg of animal per dose see above) or the vehicle and then perfused. The corpus callosum of three animals per condition was studied. For histological examination, forebrain coronal sections (20 μm) were taken of the rostral part of the corpus callosum as previously described^80^,^81^ and stained with eriochrome cyanine using a standard protocol in order to study the myelin. Briefly, cryosections on glass slides were immersed in acetone for 5 minutes, dried and submerged in eriochrome cyanine solution (Sigma) for 1 hour and then differentiated in 5% (w/v) iron alum for 5–10 minutes, followed by a second differentiation with borax-ferricyanide solution for 5 minutes. The stained area was measured with the Image J software and related to the total analyzed area. For the immunofluorescence analysis, tissue sections were stained with anti-MBP (1:200; Serotec; Cat#MCA4095) for myelin, anti-PDGFRα (1:200; Santa Cruz; Cat#sc338) for OPCs, anti-CC1 (1:100; Calbiochem, Cat#OP80) and anti-GFAP (Glial fibrillary acidic protein; 1:200; Dako; Cat#Z0334) for astrocytes, and tomato lectin (TL; 1:67; Sigma; Cat#L0651) for microglia. Optical images were acquired using an Olympus BX61 microscope and fluorescence images were taken using a DFC480 FX digital camera (Leica) coupled to a Leica DM5000 B microscope or a confocal SP5 resonant scanner (Leica). Myelin staining (with MBP and eriochrome cyanine) and CC1 staining were quantified by taking pictures of non-overlapping fields of coronal sections of the corpus callosum, between the lateral ventricles (2 fields per section, 5–6 sections of each mice; using a x20 objective) and by measuring the fluorescence intensity or blue staining using the Image J software. The number of OPCs per field was also counted in pictures of the midline of the corpus callosum using the x40 objective (4–6 sections per mice). Astrocytes and microglia were evaluated in pictures of the same area of the corpus callosum using the x40 objective and quantified by measuring the area occupied by GFAP or TL staining respectively using the Image J software (4–6 sections per mice). Data were expressed as the mean ± SEM.

### *In vivo* remyelination assay in LPC demyelination model

Anaesthetized 8 week old C57/BL6 male mice were injected with 2 μl of 1% LPC through a hole drilled in the skull at stereotactic coordinates 1.2 mm posterior, 0.5 mm lateral, 1.4 mm deep to the bregma over 4 minutes using a 30-gauge needle attached to a Hamilton syringe, driven by a KD Scientific Nano pump, for 4 minutes to reduce backflow as previously described^82^. At 9 days post injection (dpi), the first intraperitoneal dose of PDE7-GSK3 inhibitors (VP1.15 or VP3.15; 10 mg/kg of animal) was injected using the same solution detailed above for the cuprizone model. Each inhibitor was injected into a group of 10 mice and another group of 10 mice was injected with the vehicle. Two days after, a second dose was injected (11 dpi) and 5 mice of each group were perfused at 14 dpi with 4% PFA and 2% glutaraldehyde (GTA) in phosphate buffer. The remaining 5 animals of each group received a third injection of inhibitors (15 dpi) and were perfused at 21 dpi. A control group of 3 non-lesioned and non-treated mice was used as control. Fixed brains were post-fixed in 1% GTA for 7 days and then coronally cut into 1 mm slices. The slices containing the injected areas were selected and the area of interest was cut and processed for electron microscopy into a resin (Araldite/TAAB 812 Resin Kit, TAAB) following manufacturer instructions.

In order to detect the injured areas for investigation, sagittal semi-thin sections (1 μm) were cut using an ultramicrotome (Leica) and stained with toluidine blue. Ultra-thin sections (60 nm thick) were cut and stained in uranyl acetate and lead citrate and the ultrastructure was analysed taking images with a GatanOrius CCD camera coupled to a transmission electronic microscope (Philips CM120). The percentage of myelinated fibres and the thickness of myelin sheaths were examined. The mean percentage of myelinated fibres was measured from 10 fields per animal. Grids were overlaid onto the images and all axons intersecting with the grid lines were counted as myelinated or not^83^,^84^. The thickness of myelin around myelinated fibres was expressed as a g-ratio (axon perimeter divided by myelinated fibre perimeter) using ImageJ by tracing around a minimum of 100 individual fibres per mouse, using a pen and electronic pad (Bamboo pad, Wacom). To select the fibres randomly, a grid composed by 8 μm^2^ squares was overlaid onto the images and fibres in alternate squares were measured^83^,^84^. Data were expressed as the mean ± SEM.

### Statistical analysis

Results were analysed with SigmaStat/SigmaPlot software (Jandel Scientific, Germany). A comparative analysis was performed using a Student’s t test (or Mann–Whitney rank-sum test) or one-way ANOVA (or post-test Turkey’s multiple comparison test). All *in vitro* experiments were repeated three times (except with human samples, two independent experiments) and at least three mice per experimental group were analysed for the *in vivo* experiments. Statistical significance was set at: p < 0.05, *p < 0.05, **p < 0.01, ***p < 0.001.

## Additional Information

**How to cite this article:** Medina-Rodríguez, E. M. *et al*. Promoting *in vivo* remyelination with small molecules: a neuroreparative pharmacological treatment for Multiple Sclerosis. *Sci. Rep.* **7**, 43545; doi: 10.1038/srep43545 (2017).

**Publisher's note:** Springer Nature remains neutral with regard to jurisdictional claims in published maps and institutional affiliations.

## Supplementary Material

Supplementary Information

## Figures and Tables

**Table 1:** Compounds used in the study.

Compound Name	Chemical Structure	PDE7 inhibition IC_50_ (μM)	GSK-3 inhibition IC_50_ (μM)
VP1.15	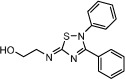	1.1	1.95
VP3.15	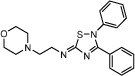	1.59	0.88
TDZD8	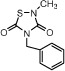	>100	0.69

Chemical structures and potency of inhibition of PDE7 and GSK3 (Modified from^33^).

**Table 2:** Pharmacokinetic parameters of VP3.15.

Route	Matrix	T_max_ (h)	C_max_ (ng.ml^−1^)	AUC_last_ (h.ng.mL^−1^)	AUC_inf_ (h.ng.mL^−1^)	MRT_last_ (h)	MRT_inf_ (h)
**VP3.15**	Plasma	0.25	1990.43	1956.74	1998.55	0.82	0.91
i.p.	Brain^a^	0.08	1982.38	1489.25	1506.00	1.21	1.25
**VP3.15**	Plasma	1.00	880.76	2292.94	2378.17	1.64	1.86
p.o	Brain^a^	0.5	496.55	1375.08	1794.28	2.07	4.01

Pharmacokinetic parameters of VP3.15 in plasma and brain following a single intraperitoneal (10 mg.kg^−1^) and oral (50 mg.kg^−1^) dose in male BALB/c mice.

^a^Brain concentration and AUC expressed as ng.g^−1^ and h.ng.g^−1^ respectively. MRT: mean residence time.
