# Supplementary material for: Promoting in vivo remyelination with small molecules: a neuroreparative pharmacological treatment for Multiple Sclerosis
Source: Sci Rep. 2017 Mar 3;7:43545. doi: 10.1038/srep43545 (PMC5335257; doi:10.1038/srep43545)
Supplement: Supplementary Information [file srep43545-s1.pdf]

Promoting *in vivo* remyelination with small molecules: a neuroreparative pharmacological treatment for Multiple Sclerosis

Authors: Eva María Medina-Rodríguez[1], Ana Bribián[1,2*],Amanda Boyd[3], Valle Palomo[4,] Jesús Pastor[5], Alfonso Lagares[6], Carmen Gil[4], Ana Martínez[4], Anna Williams[3], Fernando de Castro[1,2*]

Affiliations:

[1]Grupo de Neurobiología del Desarrollo-GNDe, Hospital Nacional de Parapléjicos, Finca la Peraleda s/n, E- 45071, Toledo, Spain.

[2]Instituto Cajal-CSIC, Avda. Dr. Arce 37, E-28002, Madrid, Spain

[3]MRC-Centre for Regenerative Medicine, University of Edinburgh, 5 Little France Drive, EH164UU, Edinburgh, UK.

[4]Centro de Investigaciones Biológicas, CIB-CSIC, Calle Ramiro de Maeztu 9, E-28040, Madrid, Spain.

[5]Servicio de Neurofisiología Clínica, Hospital La Princesa, Calle Diego de León 62, E-28006,Madrid, Spain.

[6]Servicio de Neurocirugía, Hospital 12 de Octubre, Avda. de Córdoba s/n, E-28041,Madrid, Spain.

*** Corresponding authors:**

F. de Castro PhD, MD
Grupo de Neurobiología del Desarrollo-GNDe
Instituto Cajal-CSIC
Avda. Dr. Arce, 37
E-28002-Madrid, Spain.
E-mail: fdecastro@cajal.csic.es
Phone: +34 91-5854650

Ana Bribián, PhD
Instituto Cajal-CSIC
Avda. Dr. Arce, 37
E-28002-Madrid, Spain.
E-mail: abribian@cajal.csic.es
Phone: +34 91-5854708

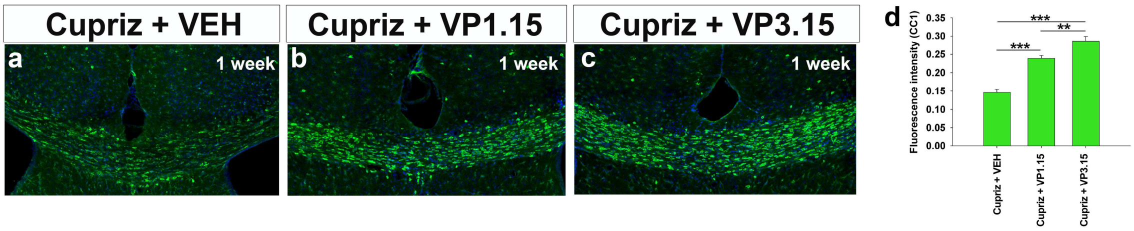

Supplementary figure 1. PDE7 inhibition increases the number of CC1 cells. (a-c) Images of vehicle, VP1.15 and VP3.15 treated animals, 1 week after cuprizone withdrawal. **(d)** Quantification of CC1 fluorescence in the corpus callosum. CC1 immunofluorescence intensity increased with PDE7 inhibitors. Values are given as mean ± SEM and the results of Student's t-test are represented as $*p < 0.05$, $**p < 0.01$, and $***p < 0.001$

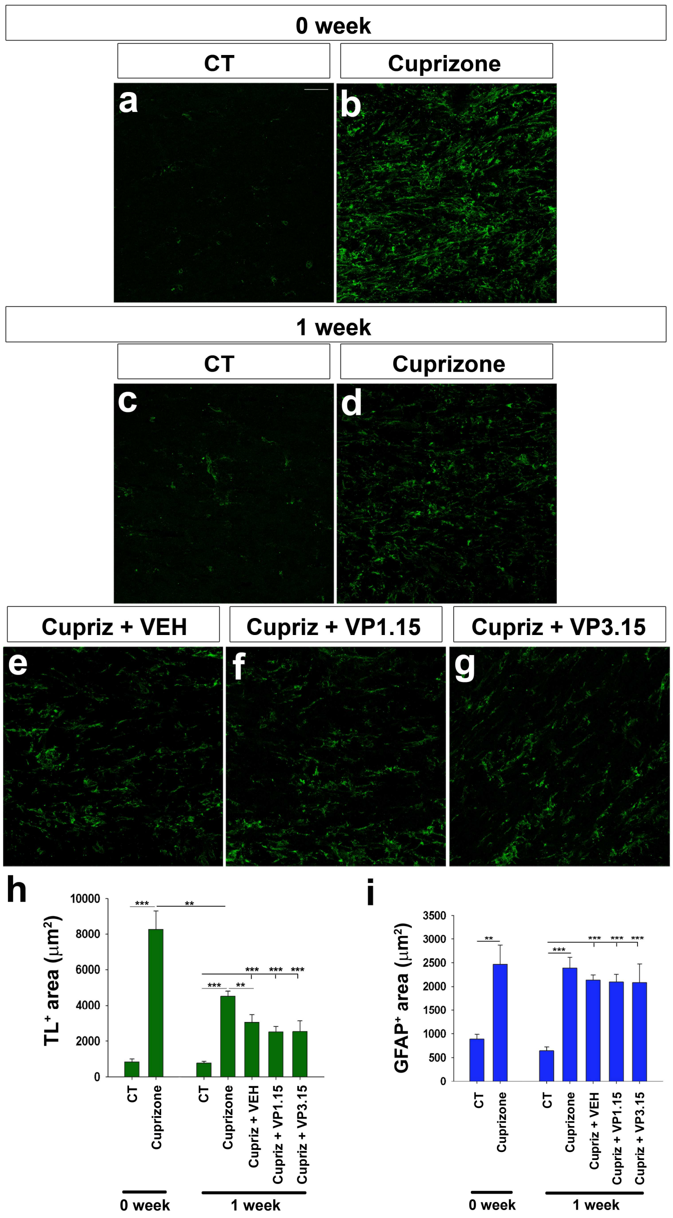

Supplementary figure 2. The number of microglia and astrocytes after cuprizone did not change in response to PDE7-GSK3 inhibition. (a-g) Immunofluorescence images showing microglia after cuprizone-induced demyelination, immediately after cuprizone withdrawal **(a, b)**, 1 week after cuprizone **(c, d)** and after alternate day treatment with PDE7-GSK3 inhibitors or vehicle during that week **(e-g). (h)** Quantification of microglial staining using pixel count of TL staining showed a strong increase in the corpus callosum after the cuprizone administration (0 week) compared to the control group. 1 week after cuprizone withdrawal, TL staining was still higher than in the control mice but lower than immediately after cuprizone withdrawal (0week). Treatment of the mice with vehicle containing DMSO led to a decrease in microglial TL staining, but treatment with VP1.15 or VP3.15 showed no additional effect over this. (i) Histogram quantifying astrocyte staining using GFAP showed an increase after cuprizone feeding in the corpus callosum compared to control mice but no changes were observed after treatment with PDE7-GSK3 inhibitors. Scale bar represents 25 µm for a-g. Values are given as mean ± SEM and the results of Student's *t*-test are represented as *p < 0.05, **p < 0.01, and ***p < 0.001
